# Supplementary material for: Discovery of a novel cardiac-specific myosin modulator using artificial intelligence-based virtual screening
Source: Nat Commun. 2023 Nov 24;14:7692. doi: 10.1038/s41467-023-43538-y (PMC10673995; doi:10.1038/s41467-023-43538-y)
Supplement: Supplementary file 1 — Supplementary Information [file 41467_2023_43538_MOESM1_ESM.pdf]

Supplementary Information

Discovery of a novel cardiac-specific myosin modulator using artificial intelligence-based virtual screening

Priyanka Parijat[1], Seetharamaiah Atilli[1], Zoe Hoare[2], Michael Shattock[2], Victor Kenyon[3] and Thomas Kampourakis[1,*]

[1]Randall Centre for Cell and Molecular Biophysics; and British Heart Foundation Centre of Research Excellence, King's College London, London, SE1 1UL, United Kingdom

[2]School of Cardiovascular and Metabolic Medicine and Sciences; Rayne Institute and British Heart Foundation Centre of Research Excellence, King's College London, London, SE5 9NU, United Kingdom

[3]Atomwise Inc., San Francisco, California, USA

Supplementary Information Figures and Figure Legends

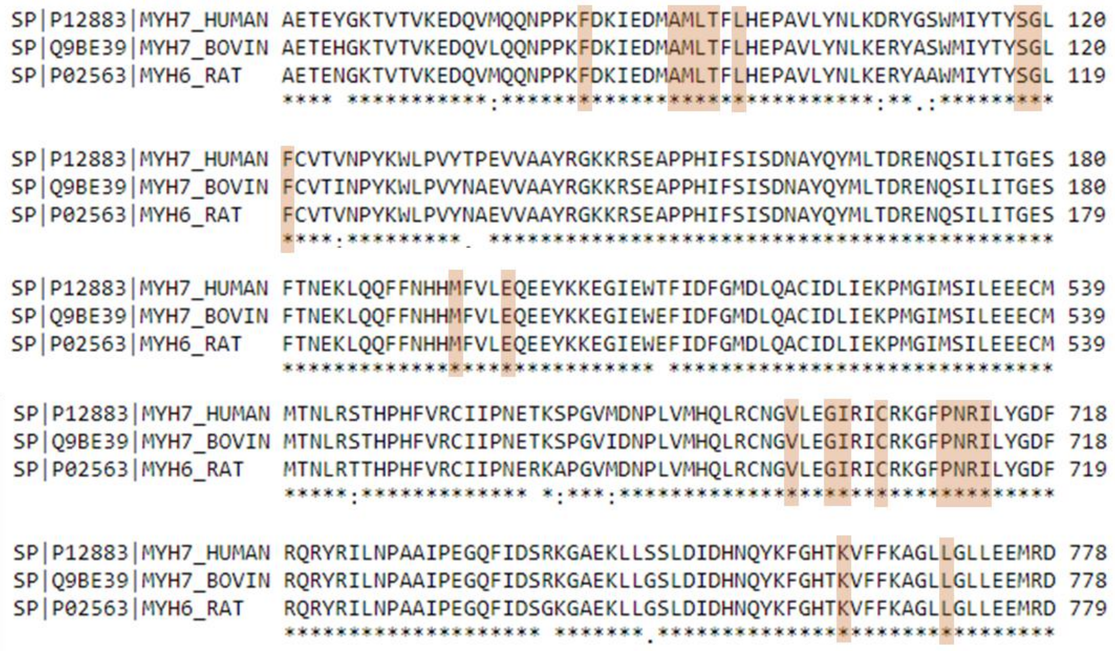

Supplementary Figure 1. Sequence alignment of human, bovine and rat cardiac myosin motor domain spanning residues 1-779. Residues forming the Omecamtiv Mecarbil-binding site in PDB 4PA0 are highlighted in orange.

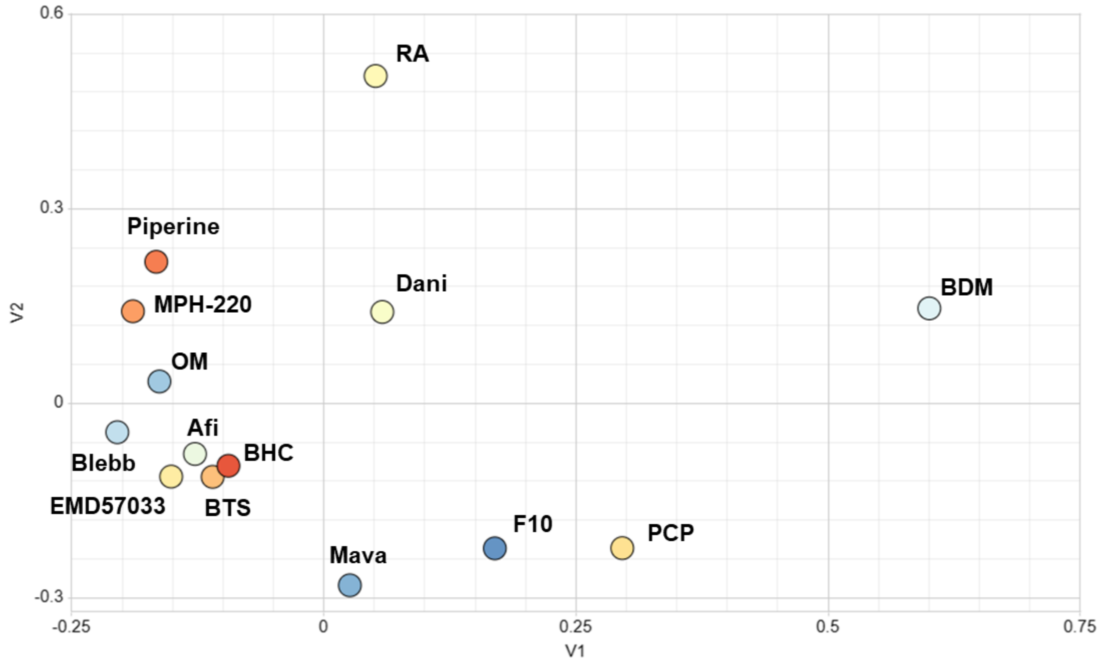

Supplementary Figure 2. Structural clustering of known small molecule myosin modulators using multi-dimensional scaling in ChemMineTools[1]. RA – retinoic acid; Dani – Danicamtiv; OM – Omecamtiv Mercarbil; Blebb – Blebbistatin; Afi – Aficamten; BTS - N-benzyl-p-toluene sulphonamide; Mava – Mavacamten; PCP – Pentaclorpseudiline; BHC - -((N-butylethanimidoyl)ethyl)-4-hydroxy-2H-chromen-2-one; BDM – 2,3 – Butadione monoxime.

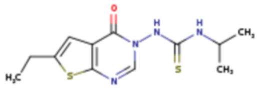

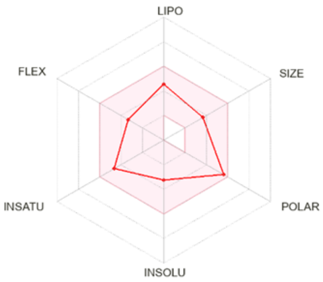

SMILES CCc1sc2c(c1)c(=O)n(cn2)NC(=S)NC(C)C

Physicochemical Properties	
Formula	C12H16N4OS2
Molecular weight	296.41 g/mol
Num. heavy atoms	19
Num. arom. heavy atoms	9
Fraction Csp3	0.42
Num. rotatable bonds	5
Num. H-bond acceptors	2
Num. H-bond donors	2
Molar Refractivity	83.71
TPSA	119.28 Å²

Lipophilicity	
Log $P_{o/w}$ (iLOGP)	2.53
Log $P_{o/w}$ (XLOGP3)	2.47
Log $P_{o/w}$ (WLOGP)	1.66
Log $P_{o/w}$ (MLOGP)	1.68
Log $P_{o/w}$ (SILICOS-IT)	3.28
Consensus Log $P_{o/w}$	2.32

Water Solubility	
Log *S* (ESOL)	-3.25
Solubility	1.65e-01 mg/ml ; 5.57e-04 mol/l
Class	Soluble
Log *S* (Ali)	-4.62
Solubility	7.12e-03 mg/ml ; 2.40e-05 mol/l
Class	Moderately soluble
Log *S* (SILICOS-IT)	-3.54
Solubility	8.53e-02 mg/ml ; 2.88e-04 mol/l
Class	Soluble

Pharmacokinetics	
GI absorption	High
BBB permeant	No
P-gp substrate	No
CYP1A2 inhibitor	Yes
CYP2C19 inhibitor	Yes
CYP2C9 inhibitor	Yes
CYP2D6 inhibitor	No
CYP3A4 inhibitor	No
Log K_p (skin permeation)	-6.35 cm/s

Druglikeness	
Lipinski	Yes; 0 violation
Ghose	Yes
Veber	Yes
Egan	Yes
Muegge	Yes
Bioavailability Score	0.55

Medicinal Chemistry	
PAINS	0 alert
Brenk	1 alert: thiocarbonyl_group
Leadlikeness	Yes
Synthetic accessibility	3.33

Supplementary Figure 3. Predicted ADMET properties of F10 using SwissADME[2].

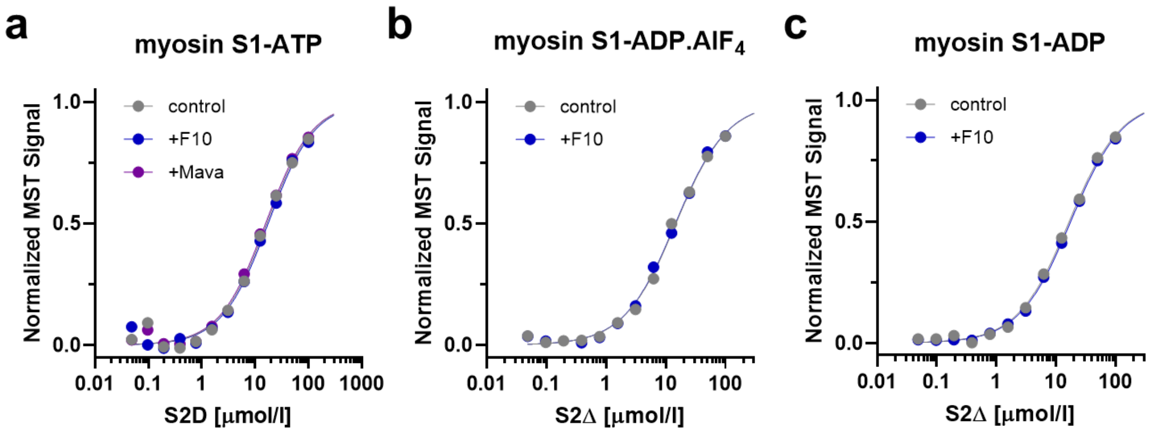

Supplementary Figure 4. Normalized MST binding curves for Alexa647-labelled bovine β-cardiac myosin S1 titrated against increasing concentrations of myosin S2Δ in the absence (grey) and in the presence of either Mava (purple) or F10 (blue). Binding experiments were performed in the presence of 1 mmol L^{-1} (a) ATP, (b) $ADP.AlF_4$ or (c) ADP.

Source data are provided as a Source Data file.

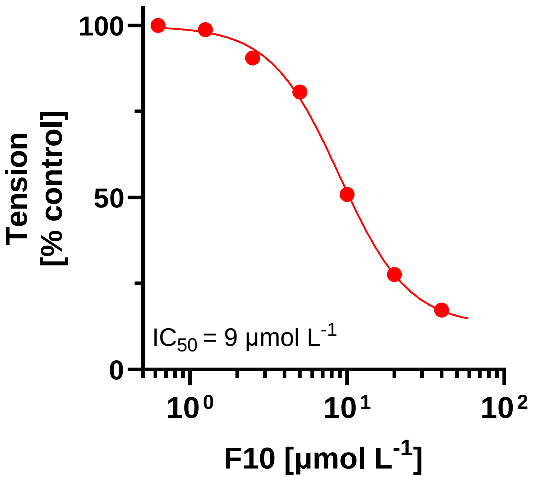

Supplementary Figure 5. Normalized dose-response curve for the effect of F10 on active isometric force at pCa 5.9 (corresponding to about 50% maximal activation).

Source data are provided as a Source Data file.

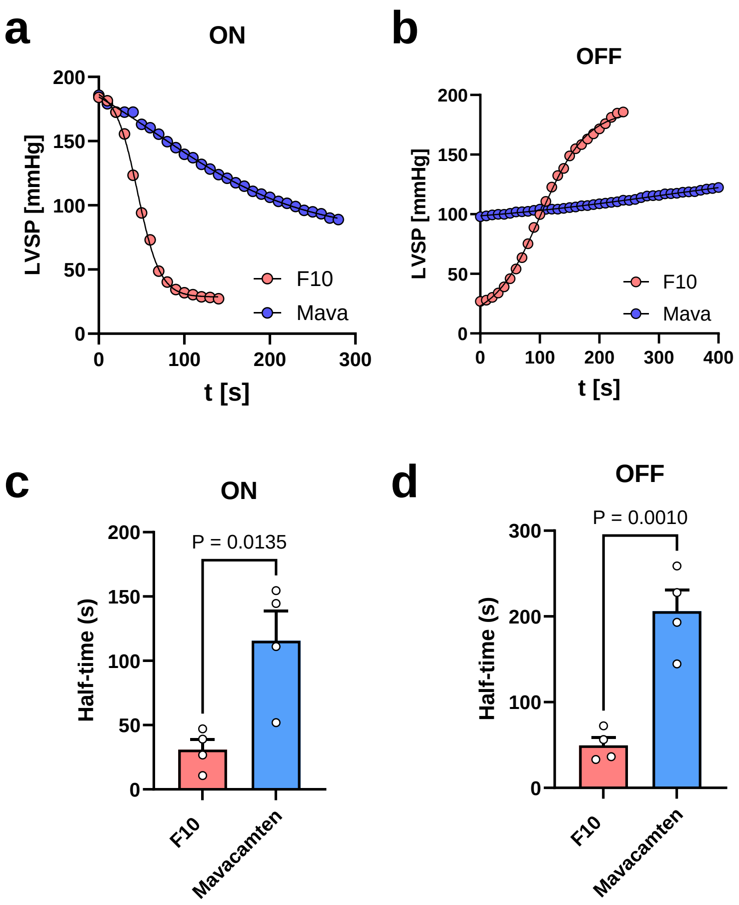

Supplementary Figure 6. Representative traces of left ventricular systolic (LVSP) during (a) perfusion of rat hearts with Krebs-Henseleit solution containing either F10 (red) or Mavacamten (blue), and (b) after washout. Continuous lines represent fit of data points to a Boltzmann sigmoidal function (LVSP=t+(Top-Bottom)/(1+exp((half-time-t)/Slope))). Similar fits were applied to each individual heart and the extracted half-times for the ON and OFF rates were averaged and are shown in (c) and (d), respectively. In this fit, ‘Top’ was constrained to being equal to the steady-state LVSP immediately prior to drug application. Means ± s.e.m., n=4 rat hearts. Statistical significance of differences between F10 and Mavacamten were assessed with two-tailed, unpaired student’s t-test.

Source data are provided as a Source Data file.

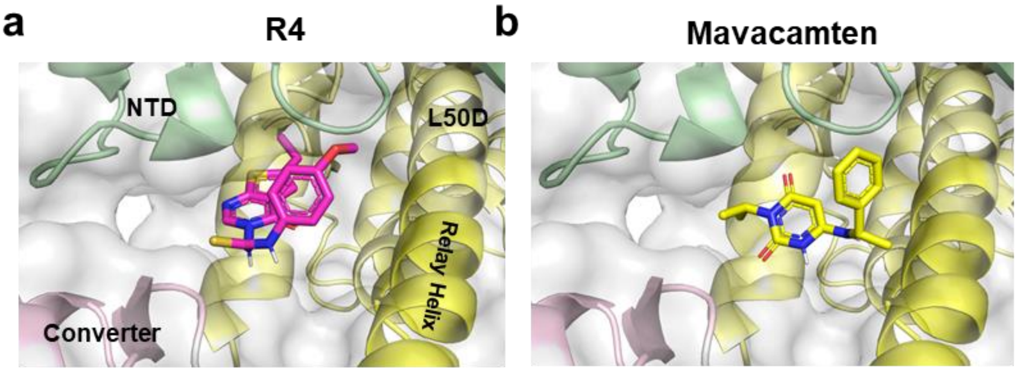

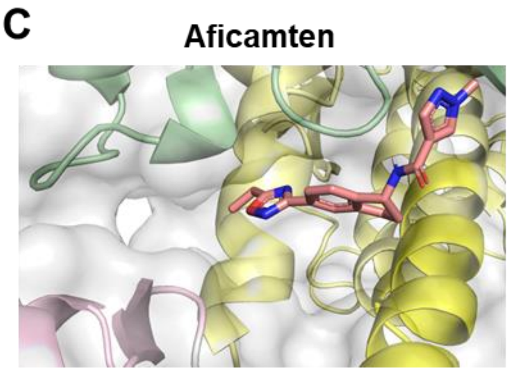

d

compound	ΔG [kcal mol^{-1}]	K_d [μmol L^{-1}]
F10	-6.2	28.4
R4	-6.5	17.3
Mavacamten	-6.6	14.4
Aficamten	-7.0	7.4

Supplementary Figure 7. Top scoring docking poses predicted by AutoDock Vina for (a) F10-derivative R4 (purple), (b) Mavacamten (yellow) and (c) Aficamten (pink) in the OM-binding pocket of human cardiac myosin S1. The N-terminal domain (NTD, green), converter (pink) and lower 50 ka domain (L50D, yellow) are labelled accordingly. (d) Calculated changes in free energy (ΔG) and steady dissociation constant K_d.

Supplementary Information Tables and Table Legends

Supplementary Table S1. Summary of bi-exponential fits to mant-ATP chase experiments in bovine β-cardiac myosin S1, synthetic thick filament and cardiac myofibrils.

	Slow Phase [%]	k_{fast} [s^{-1}]	k_{slow} [s^{-1}]
**β-cardiac myosin S1**			
control (n=5)	21.4 ± 1.8	0.0496 ± 0.0100	0.0058 ± 0.0010
+Mava (n=6)	71.0 ± 5.5 (*P<0.0001)	0.0251 ± 0.0102 (*P=0.1672)	0.0035 ± 0.0002 (*P=0.0264)
+F10 (n=6)	69.8 ± 5.1 (*P<0.001)	0.0454 ± 0.0057 (*P=0.9424)	0.0014 ± 0.0002 (*P<0.0001, $P=0.0292)
**Synthetic Myosin Filaments**			
Control (n=5)	22.9 ± 2.8	0.0769 ± 0.0163	0.0046 ± 0.0007
+Mava (n=7)	71.2 ± 2.4 (*P<0.0001)	0.0593 ± 0.0188^{ns} (*P=0.9270)	0.0033 ± 0.0007 (*P=0.3223)
+F10 (n=7)	63.2 ± 4.2 (*P<0.0001)	0.0784 ± 0.0208 (*P=0.9528)	0.0027 ± 0.0005 (*P=0.2187)
**Cardiac myofibrils**			
control (n=5)	25.4 ± 0.2	0.0719 ± 0.0193	0.0053 ± 0.0009
+Mava (n=6)	46.6 ± 8.0 (*P<0.0285)	0.0637 ± 0.0271 (*P=0.9529)	0.0027 ± 0.0009 (*P=0.0734)
+F10 (n=6)	67.8 ± 3.6 (*P<0.0001, $P=0.0221)	0.0552 ± 0.0106 (*P=0.8080)	0.0021 ± 0.0002 (*P=0.0185)

Means s.e.m. with the number of independent experiments (n) shown for each condition. Statistical significance of differences between values was assessed with a one-way ANOVA followed by Tukey's post-hoc test. P-values are shown in brackets with *P for F10 or Mava vs control, and $P for Mava vs F10. Source data are provided as a Source Data file.

Supplementary Table S2. Summary for the effect of F10 on rat ventricular trabeculae mechanics and fluorescence polarization.

	control	F10	P value
**T_{min} (mN mm^{-2})**	3.4 ± 0.5	3.5 ± 0.6	0.8561
**T_{max} (mN mm^{-2})**	69.6 ± 2.6	15.6 ± 1.8	<0.0001
**$\langle P_2 \rangle_{min}$**	0.144 ± 0.003	0.193 ± 0.009	0.0319
**$\langle P_2 \rangle_{max}$**	0.078 ± 0.008	0.207 ± 0.023	0.0147
**pCa_{50}**	5.88 ± 0.02	5.69 ± 0.01	0.0137
**n_H**	7.60 ± 0.05	5.57 ± 0.33	0.0205
**k_{tr} [s^{-1}]**	12.0 ± 1.9	35.1 ± 1.3	0.0056

Means s.e.m., n=3-5 trabeculae experiments. Statistical significance of differences between control and F10 treatment was assessed with a two-tailed, paired student's t-test.

Source data are provided as a Source Data file.

Supplementary Table S3. Summary for the effects of 20 μmol L^{-1} F10 on haemodynamic parameters of Langendorff-perfused rat hearts.

	Baseline	20 μmol L^{-1} F10	P value
**LVSP [mmHg]**	168.0 ± 8.5	58.3 ± 2.2	0.0016
**LVEDP [mmHg]**	1.9 ± 0.2	22.0 ± 1.1	0.0046
**dP dt^{-1}_{max} [mmHg s^{-1}]**	3978.8 ± 108.0	1329.0 ± 153.8	0.0004
**dP dt^{-1}_{min} [mmHg s^{-1}]**	-3352.4 ± 134.4	-1300.0 ± 104.2	0.0014
**Heart rate [bpm]**	303 ± 8	300 ± 4	0.7277
**Coronary flow [ml min^{-1}]**	15.6 ± 1.7	16.7 ± 2.3	0.6643

Means ± s.e.m. for n = 4 rat hearts. Statistical significance of differences between baseline and F10 was assessed with a two-tailed, paired student's t-test.

Source data are provided as a Source Data file.

Supplementary Information References

1 Backman, T. W., Cao, Y. & Girke, T. ChemMine tools: an online service for analyzing and clustering small molecules. *Nucleic Acids Res* **39**, W486-491, doi:10.1093/nar/gkr320 (2011).

2 Daina, A., Michielin, O. & Zoete, V. SwissADME: a free web tool to evaluate pharmacokinetics, drug-likeness and medicinal chemistry friendliness of small molecules. *Sci Rep* **7**, 42717, doi:10.1038/srep42717 (2017).
