# Supplementary material for: Discovery of a novel cardiac-specific myosin modulator using artificial intelligence-based virtual screening
Source: Nat Commun. 2023 Nov 24;14:7692. doi: 10.1038/s41467-023-43538-y (PMC10673995; doi:10.1038/s41467-023-43538-y)
Supplement: Supplementary file 3 — Reporting Summary [file 41467_2023_43538_MOESM3_ESM.pdf]

nature portfolio

Corresponding author(s): Thomas Kampourakis

Last updated by author(s): Oct 17, 2023

# Reporting Summary

Nature Portfolio wishes to improve the reproducibility of the work that we publish. This form provides structure for consistency and transparency in reporting. For further information on Nature Portfolio policies, see our Editorial Policies and the Editorial Policy Checklist.

## Statistics

For all statistical analyses, confirm that the following items are present in the figure legend, table legend, main text, or Methods section.

| n/a | Confirmed | |
|---|---|---|
| ☐ | ☒ | The exact sample size (*n*) for each experimental group/condition, given as a discrete number and unit of measurement |
| ☐ | ☒ | A statement on whether measurements were taken from distinct samples or whether the same sample was measured repeatedly |
| ☐ | ☒ | The statistical test(s) used AND whether they are one- or two-sided<br>*Only common tests should be described solely by name; describe more complex techniques in the Methods section.* |
| ☒ | ☐ | A description of all covariates tested |
| ☒ | ☐ | A description of any assumptions or corrections, such as tests of normality and adjustment for multiple comparisons |
| ☐ | ☒ | A full description of the statistical parameters including central tendency (e.g. means) or other basic estimates (e.g. regression coefficient) AND variation (e.g. standard deviation) or associated estimates of uncertainty (e.g. confidence intervals) |
| ☐ | ☒ | For null hypothesis testing, the test statistic (e.g. *F*, *t*, *r*) with confidence intervals, effect sizes, degrees of freedom and *P* value noted<br>*Give P values as exact values whenever suitable.* |
| ☒ | ☐ | For Bayesian analysis, information on the choice of priors and Markov chain Monte Carlo settings |
| ☒ | ☐ | For hierarchical and complex designs, identification of the appropriate level for tests and full reporting of outcomes |
| ☒ | ☐ | Estimates of effect sizes (e.g. Cohen's *d*, Pearson's *r*), indicating how they were calculated |

*Our web collection on statistics for biologists contains articles on many of the points above.*

## Software and code

Policy information about availability of computer code

| | |
|---|---|
| Data collection | Dedicated program written in LabView 2014 (National Instruments), LabChart 8 (AD instruments), BMG Labtech Reader Control Software V6.20 |
| Data analysis | Microsoft Excel for Microsoft 365 MSO (Version 2308 Build 16.0), GraphpPad Prism 10, LabChart 8, Pymol 2.4.1, MARS Data Analysis Software (BMG Labtech) |

For manuscripts utilizing custom algorithms or software that are central to the research but not yet described in published literature, software must be made available to editors and reviewers. We strongly encourage code deposition in a community repository (e.g. GitHub). See the Nature Portfolio guidelines for submitting code & software for further information.

## Data

Policy information about availability of data

All manuscripts must include a data availability statement. This statement should provide the following information, where applicable:

- Accession codes, unique identifiers, or web links for publicly available datasets
- A description of any restrictions on data availability
- For clinical datasets or third party data, please ensure that the statement adheres to our policy

The data supporting the findings of the study are available in the article and its Supplementary Information. The data generated in this study are provided in the Source Data file. The source data for Figures 2, 3, 4, 5 and 6, Supplementary Figures 4, 5 and 6, and Supplementary Tables 1, 2 and 3 are available with this paper.

The structure of Omecamtiv Mecarbil bound to human -cardiac myosin (PDB code 4PA0) was retrieved from Protein Data Bank (https://www.rcsb.org/structure/4pa0).

## Research involving human participants, their data, or biological material

Policy information about studies with human participants or human data. See also policy information about sex, gender (identity/presentation), and sexual orientation and race, ethnicity and racism.

| | |
|---|---|
| Reporting on sex and gender | N/A |
| Reporting on race, ethnicity, or other socially relevant groupings | N/A |
| Population characteristics | N/A |
| Recruitment | N/A |
| Ethics oversight | N/A |

Note that full information on the approval of the study protocol must also be provided in the manuscript.

# Field-specific reporting

Please select the one below that is the best fit for your research. If you are not sure, read the appropriate sections before making your selection.

☒ Life sciences ☐ Behavioural & social sciences ☐ Ecological, evolutionary & environmental sciences

For a reference copy of the document with all sections, see nature.com/documents/nr-reporting-summary-flat.pdf

# Life sciences study design

All studies must disclose on these points even when the disclosure is negative.

| | |
|---|---|
| Sample size | No sample size calculations were performed. The sample size used for each experiment was chose to be statistically adequate to describe and interpret the results while minimizing the number of animals used in the study for the relevant experiments (in accordance with the 3Rs of animal welfare). |
| Data exclusions | No data were excluded from analysis. |
| Replication | All experiments included in the paper were repeated multiple times. All replicates are biological repeats (i.e. each measurement corresponds to a different biological preparation) except where stated otherwise. All attempts at replicating the data were successful. The exact number of independent repeats for each experiment are shown in the corresponding figure legends, supplementary information figures and tables, and source data file. |
| Randomization | Samples were allocated randomly. |
| Blinding | Top scoring compounds from the virtual high throughput screen were tested in a blinded biochemical counter-screen with the compound identities and structures being revealed after the completed data set was returned to Atomwise Inc. Further blinding was not relevant to current study focusing on characterizing the biochemical and biophysical features of the identified active hit compound. |

# Reporting for specific materials, systems and methods

We require information from authors about some types of materials, experimental systems and methods used in many studies. Here, indicate whether each material, system or method listed is relevant to your study. If you are not sure if a list item applies to your research, read the appropriate section before selecting a response.

## Materials & experimental systems

| n/a | Involved in the study |
| --- | --- |
| ☒ | ☐ Antibodies |
| ☒ | ☐ Eukaryotic cell lines |
| ☒ | ☐ Palaeontology and archaeology |
| ☐ | ☒ Animals and other organisms |
| ☒ | ☐ Clinical data |
| ☒ | ☐ Dual use research of concern |
| ☒ | ☐ Plants |

## Methods

| n/a | Involved in the study |
| --- | --- |
| ☒ | ☐ ChIP-seq |
| ☒ | ☐ Flow cytometry |
| ☒ | ☐ MRI-based neuroimaging |

# Animals and other research organisms

Policy information about studies involving animals; ARRIVE guidelines recommended for reporting animal research, and Sex and Gender in Research

| | |
| --- | --- |
| Laboratory animals | Wistar rats, 200-250g, 7 weeks old |
| Wild animals | No wild animals were used in this study. |
| Reporting on sex | Only male Wistar rats were used for the experiments to avoid compounding effects of physiological hypertrophy (i.e. during pregnancy, etc) on heart muscle function. No statistical analysis regarding the effect of sex was performed. |
| Field-collected samples | No filed-collected samples were used in this study. |
| Ethics oversight | No ethical approval was required for the current study. All animals were treated in accordance with the guidelines approved by the UK Animal Scientific procedures Act (1986) and European Union Directive 2010/63/EU. All procedures were performed according to Schedule 1 of the UK Animal Scientific Procedure Act, 1986, which do not require ethical approval. All procedures complied with the relevant ethical regulations and were carried out in accordance with the guidelines of the Animal Welfare and Ethical Review Body (AWERB, King's College London). |

Note that full information on the approval of the study protocol must also be provided in the manuscript.
